# Sonographic diagnosis of fetal eye anomalies and their association with syndromal diseases: A retrospective multicenter analysis of 264 cases

**DOI:** 10.1111/aogs.15085

**Published:** 2025-03-04

**Authors:** Jorge Jiménez Cruz, Paul Böckenhoff, Laura Tascón Padrón, Norah Emrich, Philipp Kosian, Brigitte Strizek, Cristoph Berg, Eva Weber, Ulrich Gembruch, Annegret Geipel

**Affiliations:** ^1^ Department of Obstetrics and Prenatal Medicine Bonn University Hospital Bonn Germany; ^2^ Department of Obstetrics and Prenatal Medicine Cologne University Hospital Cologne Germany

**Keywords:** fetal eye, fetal syndromes, fetal ultrasound, ocular malformations, orbital malformations

## Abstract

**Introduction:**

This study aims to systematically describe eye malformations and correlate these with extraocular findings. Based on these findings, we propose a protocol for ultrasound evaluation of the fetal eye.

**Material and Methods:**

In this multicentric retrospective cohort study, 264 fetuses with ocular malformations from two tertiary referral centers for prenatal medicine were analyzed. Anophthalmia, microphthalmia, exophthalmos, hyper‐ or hypotelorism, cataract, aphakia, cyclopia, and retinal detachment were assessed, and their association with extraocular findings and genetic changes was investigated.

**Results:**

The majority of the cases (99.2%) were non‐isolated and presented further extraocular findings. Most commonly, the brain and central nervous system (65.9%), the limbs and the heart (46.6% each) and the cranial anatomy (41.2%) were affected. Significant associations were found between exophthalmos and anomalies of the fetal skeletal system (OR = 4.8, 95% CI 1.6–14) and cranial malformations (OR = 3.3, 95% CI 1.5–7.4). Hypotelorism showed an increased risk of cardiac anomalies (OR = 1.8, 95% CI 1.1–3.5) and brain malformations (OR = 2.16, 95% CI 1.2–4.1), with holoprosencephaly being the most common one. Fetuses with microphthalmia were more likely to have anomalies in the renal system (OR = 2.3, 95% CI 1.2–4.3). In 51.4% of the cases, a genetic aberration could be found, among them most frequently trisomy 13.

**Conclusions:**

There is a significant association between specific fetal eye anomalies and certain extraocular anomalies, as well as genetic changes. Systematic evaluation of the eye using the proposed protocol is simple to learn and highly reproducible and could help to concentrate diagnosis on a certain group of malformations. Data from this study could help to develop targeted diagnostic molecular tools.

## Graphical abstract

Finding ocular abnormalities in fetal scans should lead to an exhaustive exploration of the fetus. Including evaluation of the fetal eye in standard first and second‐trimester ultrasound protocols should be assessed.

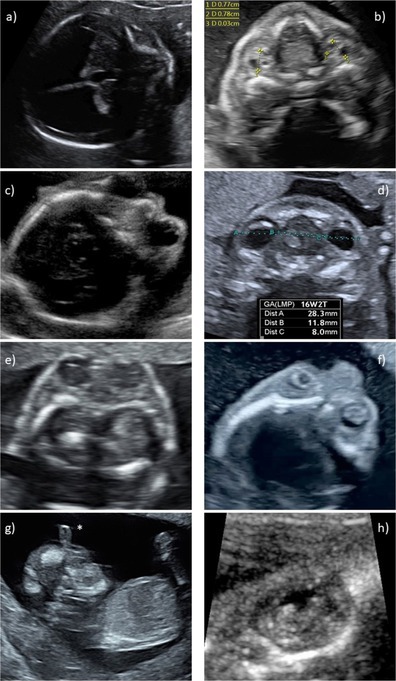

**Abbreviations**
CMA: chromosome micro arrays
OR: odds ratio

**Key message**

In this large study, the authors describe the associations between ocular findings and extraocular and genetic abnormalities. The authors propose a standardized protocol for the ultrasound scanning of the fetal eye and provide targets for molecular diagnostic tools.

## 1 INTRODUCTION

Malformations of the fetal eyes are quite rare and commonly related to other malformations as a part of more complex or syndromal disorders. Sonographic assessment of fetal eyes seems simple to learn, reproducible, and should be performed in a standardized way.^1^, ^2^ Eye form and size,^3^ eye placement in the orbit, content of the orbit, and space between both orbits have been assessed, and reference curves have been defined.^4^, ^5^, ^6^, ^7^, ^8^, ^9^ However, a systematic analysis of anomalies of the fetal eyes in a larger cohort and their association with other findings is missing. In this regard, consistent protocols and guidelines for ultrasound examination of the fetal eye have not been established.^10^, ^11^

The aim of this study is a systematic description of eye malformations in a large cohort of fetuses and their correlation with extraocular findings. Furthermore, we propose a protocol for ultrasound evaluation of the fetal eye.

## 2 MATERIAL AND METHODS

In this multicenter retrospective cohort study, we reviewed all cases with abnormalities of the fetal eyes from September 1998 to January 2020, using our computerized perinatal database. All patients were referred for specialized prenatal ultrasound examination to the Departments of Obstetrics and Prenatal Medicine at the University Hospitals of Bonn and Cologne. Both centers operate as tertiary referral centers for prenatal medicine. Trained specialists performed the ultrasound examinations in a standardized manner. Evaluation of the fetal face in axial and sagittal planes with anterior imaging of the fetal eyes is part of the routine screening protocol. In addition, a frontal tangential coronal view for assessment of the nose, lips, and nostrils and a transversal view of the head through the orbits with demonstration of the fetal lenses are routinely included. Measurements of the interorbital or biorbital distances were performed at the discretion of the specialist. All patients included have given informed consent for data collection and analysis and their use for research, as this is standard in our institution.

**FIGURE 1:**
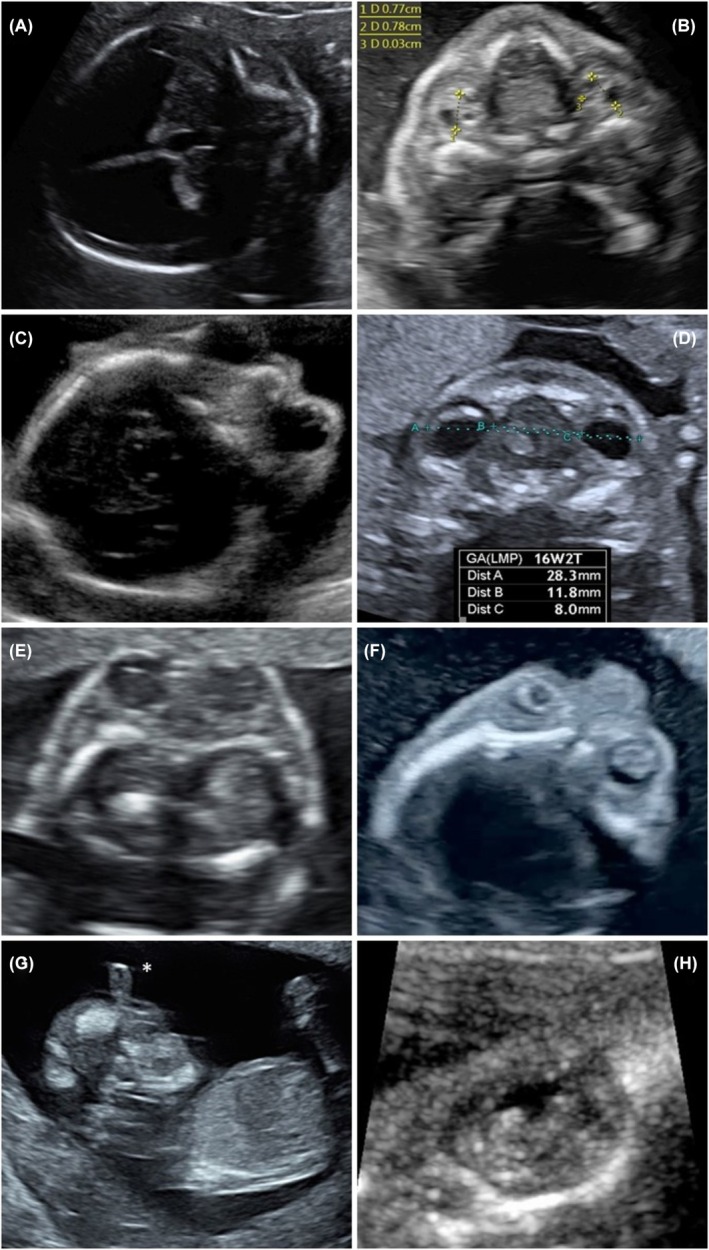
Examples of ocular findings in ultrasound. (A) anophthalmia, (B) microphthalmia, (C) exophthalmos, (D) hypertelorism, (E) hypotelorism, (F) cataract, (G) cyclopia (with proboscis*), (H) retinal detachment.

Ocular anomalies were defined as follows: anophthalmia as the absence of one or both ocular globes (Figure 1A), microphthalmia as an eye globe with an axial length being two standard deviations below the normal range^6^ (Figure 1B), exophthalmos as an abnormal protrusion of the eyeball outside the orbital cavity (Figure 1C); hyper‐ or hypotelorism was considered when interorbital distance was >95th or <5th percentile for gestational age^6^ (Figure 1D,E respectively) Cataract was described as solid, echogenic discs or areas of echogenicity within an echolucent orbit (Figure 1F). Aphakia, cyclopia (Figure 1G) and retinal detachment (Figure 1H) were considered descriptive diagnoses. Postnatal confirmation of prenatal diagnoses varied depending on the fetal outcome. For fetuses after termination or intrauterine death, postmortem examinations were limited to clinical exploration and photographic documentation; only 3 complete autopsies were performed. On living cases, postnatal pediatric exploration reports were checked to confirm the diagnosis. Only confirmed cases were included in this study.

Data from scans, demographic data, and medical history were standardized and stored in the prenatal database (ViewPoint 5.6.23.59; ViewPoint Bildverarbeitung GmbH, Weßling, Germany). Pregnancy outcomes were obtained from our perinatal database, neonatal records, or autopsy findings. Outcomes were classified as live birth, termination of pregnancy, stillbirth, and neonatal death, defined as death within the first 28 days of life.

Genetic counseling and invasive fetal testing were offered to all patients. As techniques and possibilities of genetic diagnosis have evolved over the course of the study period, only a part of the patients had access to modern diagnostic tools such as clinical exome or microarray analyses.

Statistical evaluation was carried out using SPSS version 21 (SPSS Inc. Chicago, IL). For descriptive statistics, frequencies and percentages were reported. For comparison of binomial variables, Chi squares and odds ratio (OR) with 95% confidence interval (CI) were used. Statistical significance was assumed if alpha error was lower than 5% (*p* < 0.05).

## 3 RESULTS

In total, 264 fetuses with ocular anomalies were found in our database. The mean gestational age at diagnosis was 22.4 weeks (standard deviation 6.1). The distribution of ocular deformities is presented in Table 1. Most frequently, we observed hypotelorism, hypertelorism, and microphthalmia. Additional malformations were diagnosed in 99.2% of the fetuses. We only found two cases with isolated ocular anomalies: one with microphthalmia and the other with bilateral cataract. Extraocular systems were affected as follows: the brain and nervous system (174 cases, 65.9%), the limbs and the heart (123 cases each, 46.6%) and the cranial anatomy (121 cases, 45.8%). Other malformations are listed in Table 2.

**TABLE 1:** Ocular deformities in 264 fetuses and associations with genetic and anatomical disorders.

Ocular deformities^a^ (*n*)	Genetic testing not available	Genetic testing available				Associated anatomic disorders^c^
		Chromosomal or genetic disorders			Normal genetic testing^b^	
		Numeric	Structural	Single gene		
Anophthalmia^d^ (19)	5	6	1	0	7 (50%)	Limbs, gastrointestinal tract
Exophthalmos (31)	4	7	2	7	11 (40.7%)	Skeletal system, cranium, chest, spinal tract
Hypertelorism (63)	7	12	7	4	33 (58.9%)	Limbs
Hypotelorism (72)	5	31	4	1	21 (36.8%)	Central nervous system, cardiac
Changes of fetal lens^e^ (20)	4	6	0	1	13 (65%)	—
Microphthalmia (66)	12	22	4	3	25 (46.3)	Renal system
Retinal detachment (7)	1	0	1	1	4 (66.7%)	—
Cyclopia (7)	2	4	0	0	1 (20.0%)	Umbilical cord

^a^ More than one malformation per fetus was possible (eg. exophthalmos and hypertelorism).

^b^ Percentages are referred to as the total of tested cases.

^c^ Systems mentioned here only if a statistically significant association was present as presented in Supporting Information Table S1.

^d^ 12 cases bilateral and 7 cases unilateral.

^e^ 3 cases of aphakia and 21 cases of cataract.

**TABLE 2:** Systems affected by extraocular defects related to genetic testing.

Systems affected	All, *n* (%)^a^	Normal genetic testing, *n* (%)^b^	Abnormal genetic testing, *n* (%)^b^
Central nervous system	174 (65.9%)	85 (60.7%)	55 (39.3%)
Cranial anatomy	121 (45.8%)	69 (71.1%)	28 (28.9%)
Craniofacial cleft	80 (30.3%)	36 (37.1%)	27 (42.9%)
Neck	24 (9.1%)	13 (61.9%)	8 (38.1%)
Chest	32 (12.1%)	19 (67.9%)	9 (32.1%)
Heart	123 (46.5%)	46 (45.1%)	56 (54.9%)
Abdominal wall	24 (9.2%)	8 (44.4%)	10 (55.6%)
Umbilical cord	45 (17%)	19 (47.5%)	21 (52.5%)
Gastrointestinal tract	35 (13.3%)	17 (54.8%)	14 (45.2%)
Renal system	60 (22.7%)	21 (19.6%)	28 (25.7%)
Spinal tract	16 (6.1%)	9 (60%)	6 (40%)
Limbs	123 (46.5%)	53 (50.9%)	51 (49.1%)
Skeletal system	17 (6.4%)	11 (78.6%)	3 (21.4%)

^a^ Percentage related to the whole cohort, as some fetuses presented more than one system affected (*n* = 264).

^b^ Percentage is based on the number of fetuses with known genetic testing in this group.

In order to correlate fetal ocular malformations with specific extraocular defects, a separate analysis of each anomaly was performed. In cases with exophthalmos (*n* = 31), a significantly higher rate of skeletal malformations (OR = 4.8, 95% CI 1.6–14), cranial malformations (OR = 3.3, 95% CI 1.5–7.4) and malformations of the chest (OR = 3.6, 95% CI 1.5–8.8) was observed. This group also presented a higher incidence of abnormalities of the spinal tract (OR = 5.3, 95% CI 1.8–15.7). Many cases were related to craniosynostosis syndromes like Crouzon or Alpert syndrome (10 cases). Exophthalmos was also associated with the highest rate of single‐gene disorders in our cohort (7/27, 25.9%), but was very rarely related to aneuploidy.

The 72 fetuses with hypotelorism showed significantly increased rates of cardiac (OR = 1.8, 95% CI 1.1–3.5) and brain malformations (OR = 2.16, 95% CI 1.2–4.1), holoprosencephaly being the most common one. In fetuses with hypotelorism, a high rate of numeric changes (31/57, 54.7%) was found. In the case of hypertelorism (*n* = 63) an increased risk of limb malformations was observed (OR = 2.1, 95% CI 1.1–3.7).

Fetuses with microphthalmia (*n* = 66) often presented with renal anomalies (OR = 2.3, 95% CI 1.2–4.3) and trisomy 13 (OR = 2.8, 95% CI 1.4–5.8). When cyclopia was described (*n* = 7), a significantly increased risk of abnormalities of the umbilical cord (OR = 6.9, 95% CI 1.5–32.1) was found.

A complete description of the results of the regression analysis is presented in Supporting Information Table S1.

**FIGURE 2:**
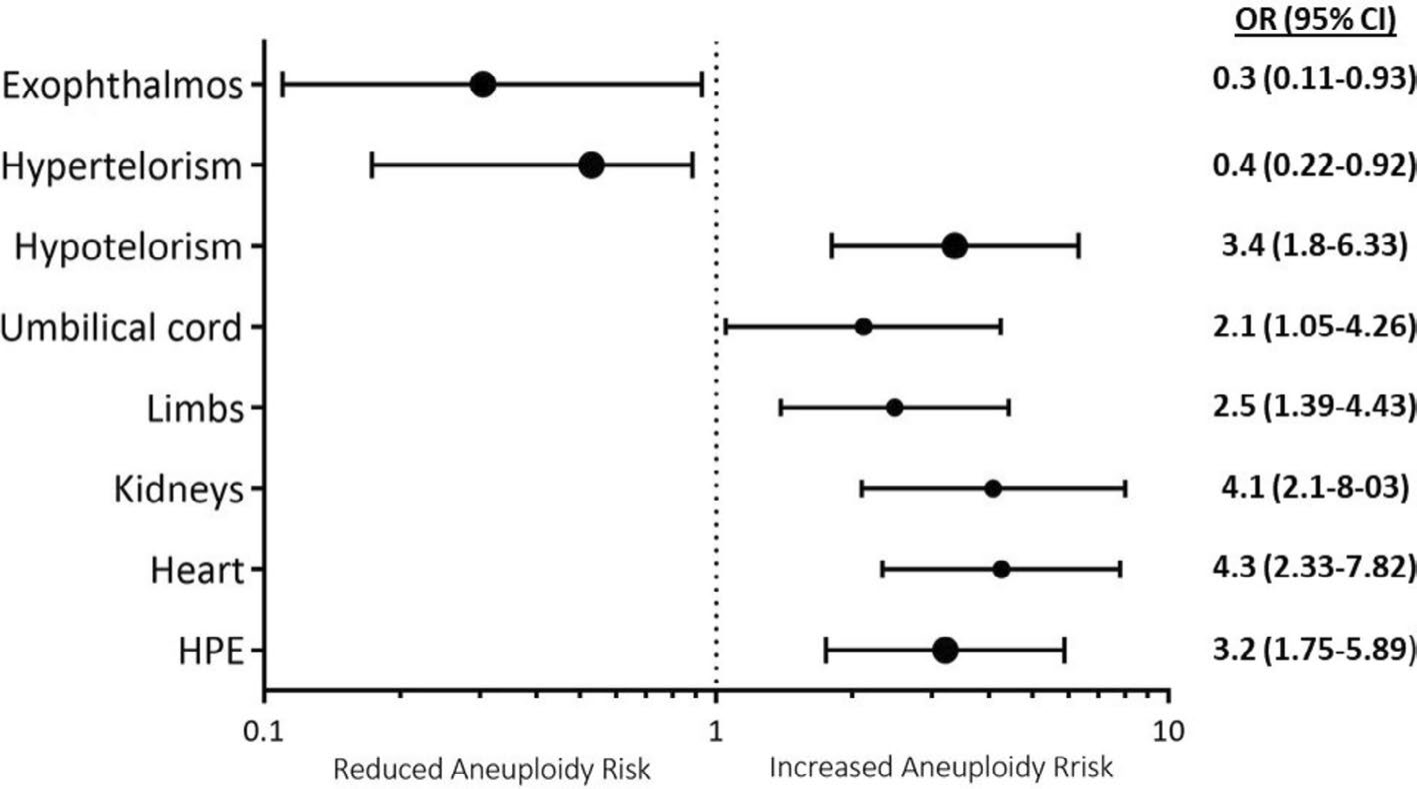
Risk of aneuploidy depending on ultrasound findings. Results presented as odds ratio and 95% confidence interval on a logarithmic scale graphic. HPE, holoprosencephaly.

A genetic analysis was available in 218 (82.6%) cases, showing no genetic or chromosomal aberrations in 106 (48.2% of all tested) fetuses. From the 112 fetuses with chromosomal or genetic disorders, 78 showed numerical aberrations, 19 presented structural genetic changes, and 15 single gene diseases. The most common chromosomal aberration was trisomy 13 in 48 cases (22% of all tested cases). Trisomy 18 was found in 12 cases (5.5%), triploidy in 11 cases (5%), deletions were described in 11 fetuses (5%) and 30 cases (13.8%) had other chromosomal aberrations like translocation, inversion, addition, etc. A complete description of the chromosomal or genetic disorders in relation to the ocular findings is presented in Table 3. The presence of extracranial findings significantly increased the risk of chromosomal or genetic disorders (OR = 3.7, 95% CI 1.7–7.9), the numerical aberrations being the most common one (aneuploidy, *n* = 67, 59.8% of all chromosomal or genetic disorders). An assessment of the risk of aneuploidy depending on ultrasound findings was performed. None of the cases with exophthalmos presented an aneuploidy, since all seven cases of numeric chromosomal aberration were caused by triploidy. Furthermore, fetuses with hypertelorism had a significantly reduced risk for aneuploidy (OR = 0.4, 95% CI 0.2–0.9), while hypotelorism significantly increased this risk (OR = 3.48, 95% CI 1.8–6.3). This increase was also observed for the combination of eye abnormalities with holoprosencephaly, limb malformations, abnormalities of the umbilical cord, or renal or cardiac anomalies (Figure 2).

**TABLE 3:** Ocular changes and associations to chromosomal or genetic disorders.

Ocular findings	Related genetic disorder (*n* = 112)
Anophthalmia	46,XY,del (13)(?q32→qter).ish, del(13)8wcp+,D13S319+,LAMP1‐) 47,XY+9(25)/46,XY (6) 47,XY+21 Trisomy 13 (*n* = 4)
Cyclopia	69,XXY Trisomy 13 (*n* = 2)
Exophthalmos	46,XX,del (5)(q34q35.3) 46,XX.del6q27,Dp11q21 69,XXX(*n* = 6) 69,XXY Heterozygous FGFR2 mutation (*n* = 7) Heterozygous KIAA0586 mutation: 46,XX,c.428delG,p.Arg143Lys
Hypertelorism	45,X0 46,XY,del (2)(q35q37.1)dn 46,XY,del(X)p22.3)lsh del(X) 46,XY,del (3)(q23q25) 46,XX,del13q22 46,XX,arr(hg19)(6q25.3‐6q23)x3(13q34)x1 46,XY.r(13)p13,q31) 69,XXX (*n* = 2) Heterozygous FGFR2 mutation: 46,XY,c.755C>G/pSer252 Heterozygous EFNB1 mutation: 46,XY,c.45,1G>A/pGly151Ser Homozygous MID1 mutation: 46,XY,tc.1102C>T/p.Arg368Ter Homozygous PLVAP mutation 46,XY,c.988C>T/p.q330* Tetrasomy 22 Trisomy 13 (*n* = 3) Trisomy 18 (*n* = 5) Unclear de novo reciprocal translocation
Hypotelorism	46,XY,del(7q32) 46,XY,del (13)(?q32‐>qter).ish del(13)8wcp+,D13S319+,LAMP1‐) 46,XY,inv. (9)(p12q13),der(16)t(6;16)(p21.3;p13.3) 47XX+9(25)/46,XX (6) 69,XXY Trisomy 13 (*n* = 22) Trisomy 18 (*n* = 7) Unclear translocation 14q18q
Microphthalmia	46,XY,der(13;14)(q10;q10),+13,inv. (9) Type I 46,XXdel (4)(p1532) 46,XX,add (17)(p1,13) 46,XY,der(13;14)(q10;q10)+13 Heterozygous ACTG1 mutation:46XY,c.98C>T(p.Ser33Phe) Homozygous ERCC5 mutation: 46,XX,c.2143C>T/p.Gin715Ter Homozygous POMT2 mutation: 46,XX,c.232G>T/p.Glu78Ter MIDAS‐Syndrome with loss of HCCS‐Gen: 46,XX,delXp22.2X22p32 Trisomy 13 (*n* = 14) Trisomy 12
Retinal detachment	Wolf‐Hirschhorn Syndrome: 46,XX,del (4)(p15,32) Homozygous FKRP Mutation: 46 XY, c.402_403delGG/p.Ala135*
Cataract	47,XX +9 (25)/46,XX (6) Trisomy 13 (*n* = 3)

The outcome could be assessed in 254 cases (96.2%). After extensive counseling, many families (172 cases, 67.7% of the cases with known outcome) opted for termination of pregnancy. In all these cases, ocular anomalies were found as a part of a syndromic constellation. Of those 82 who decided to continue pregnancy, 36 fetuses (43.9% of continued pregnancies) died in utero (*n* = 20) or within in the perinatal period (*n* = 16).

Subanalysis of continued pregnancies revealed no relation between ocular changes and the probability of prenatal death.

## 4 DISCUSSION

In this observational descriptive study, we were able to assess correlations between ocular and extraocular findings in second‐trimester sonography. To the best of our knowledge, this is the largest descriptive cohort of fetuses with ocular abnormalities published until now. In our cohort, ocular changes often correlated with defects of the central nervous system, limbs, heart, and cranial anatomy; neurological malformations being the most prevalent.

In 1991, Bronshtein et al. first described sonographic findings of fetal ocular defects.^12^ Since then, the relevant technical development of sonography allows a more precise description of those findings.^13^ Three‐dimensional ultrasound^14^ and MRI can provide additional information about related structures.^15^, ^16^ This study group also supports that the diagnosis of fetal eye malformations can be performed using standardized sonographic planes, as already described by other researchers.^11^, ^17^ In this study, the mean gestational age at diagnosis was 22 weeks. This is due to the German pregnancy care program, where second‐trimester screening is facultative at the mother's choice between the 19th and 22nd week of gestation, so probably this is the first time many malformations can be diagnosed, since the evaluation of the fetus at first trimester ultrasound only includes the evaluation of viability of the pregnancy. First trimester screening for chromosomal aberrations is facultative in Germany, and its payment is only covered for some patients at risk.

In current international standard protocols for first and second‐trimester fetal anomaly screening, only little effort is spent on detecting ocular malformations, and recommendations for scanning this area are very heterogeneous (Table 4). Evaluation of the eyes in the first trimester scan is considered facultative for the International Society of Ultrasound in Obstetrics and Gynecology (ISUOG), the German Society of Ultrasound in Medicine (DEGUM) and the American Institute of Ultrasound in Medicine (AIUM).^18^, ^19^, ^20^ The Fetal Medicine Foundation (FMF), the American College of Obstetricians and Gynecologists (ACOG), the Australasian Society of Ultrasound in Medicine (ASUM) and the Canadian Society (SOGC) do not include the exploration of the eyes in their first trimester guidelines.^21^, ^22^ For the second‐trimester ultrasound scan, FMF, DEGUM, SOGC, and ASUM consider the evaluation of the orbits a standard structure in their recommendations,^23^, ^24^, ^25^, ^26^ while AIUM and ISUOG describe this evaluation as optional,^27^, ^28^ and ACOG does not mention the eye scan in their most recent recommendations.^29^ Regarding the most optimal plane for the exploration of the ocular anatomy, no consensus has been achieved. Only FMF and AIUM recommend specific planes for these structures.

**TABLE 4:** Current recommendations for eye scans within different ultrasound societies.

Society	1st Trimester	Midtrimester	Recommended plane
ISUOG	Optional	If feasible	—
DEGUM	Optional	Standard	—
FMF London	—	Standard	Sagittal, transverse and coronal
AIUM	If suspicious	Optional	Axial and coronal
ACOG	—	—	—
ASUM	—	Standard	—
SOGC	—	Standard	—

Abbreviations: ACOG, American College of Obstetricians and Gynecologists; AIUM, American Institute of Ultrasound in Medicine; ASUM, Australasian Society for Ultrasound in Medicine; DEGUM, Deutsche Gesellschaft für Ultraschall in der Medizin; FMF London, Fetal Medicine Foundation; ISUOG, International Society of Ultrasound in Obstetrics & Gynecology; SOGC, Society of Obstetricians and Gynaecologists of Canada.

Chromosomal or genetic disorders were present in 60% of the fetuses in this cohort, with the most significant association being between hypotelorism and trisomy 13. Since this aneuploidy is known to be associated with craniofacial and neuronal defects,^30^ this correlation was not unexpected. Interestingly, chromosome 13 was involved in 55.3% (*n* = 42) of the cases of other genetic aberrations, including translocations, deletions, inversions, etc. Another significant correlation could be observed between exophthalmos and mutations related to skeletal anomalies in this study, especially mutations of the FGFR genes. This can be explained since these mutations are related to craniosynostosis syndromes like Crouzon, and exophthalmos is the result of cranial deformation.^31^, ^32^, ^33^ Also, fetuses affected by triploidy have been commonly described presenting an exophthalmos.^34^, ^35^ Present data are in line with this observation since seven out of eight fetuses with triploidy presented exophthalmos. One fetus with retinal detachment was diagnosed with Wolf‐Hirschhorn syndrome (microdeletion 4p16.3). This relation has been already described previously.^36^ Also, the relation between cataract and trisomy 13 observed in this cohort has been described in other studies.^37^

**FIGURE 3:**
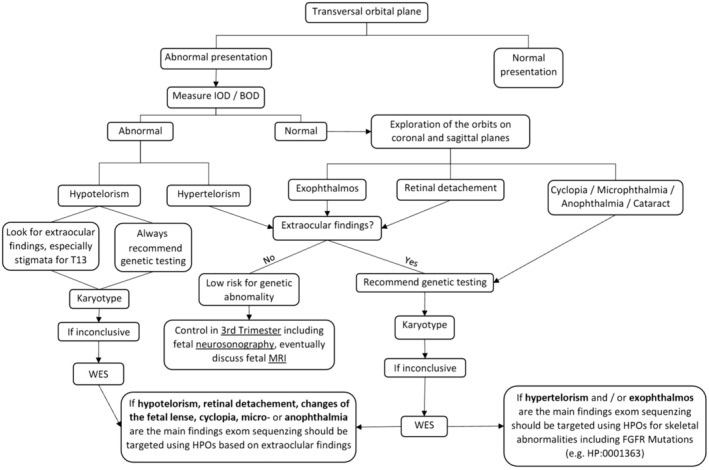
Protocol proposal for systematic evaluation of fetal eye and use of additional diagnostic tools. BOD, biorbital distance; IOD, interocular distance; HPO, human phenotype ontology; WES, whole exome sequencing.

Although only limited data about the diagnostic yield of including ocular evaluation in standard ultrasound are known, theoretical potential benefits and clinical experience based on the present data suggest that the study of the fetal eye is suitable and could improve diagnostic accuracy. This study group recommends including the evaluation of the eye in second and first‐trimester scans in standard protocols based on the demonstration of both lenses and ocular globes in a transversal presentation of the head on the orbital plane. In the case of abnormal presentation, inner and outer ocular distances should be measured, and the evaluation of the eyes in the other planes (sagittal and coronal) should be performed. An extensive scan of the fetus should be performed looking for extraocular anomalies. Based on the results of the present study, we propose a systematic protocol for the evaluation of the fetal eye and structured meaningful use of additional and invasive diagnostic tools (Figure 3). This protocol remarks that certain prenatal ocular findings should be interpreted cautiously. Isolated subtle anomalies like hypertelorism or mild exophthalmos should be considered ‘soft markers,’ warranting a detailed second‐level assessment a to exclude associated anomalies. Conversely, microphthalmia, anophthalmia, and cataracts are more definitive anomalies with significant implications. Our proposed protocol aims to strike a balance between comprehensive screening and judicious interpretation of findings, emphasizing the need for careful evaluation and follow‐up in cases of isolated subtle ocular findings.

During the study period, advancements in molecular diagnostic tools have improved the accuracy, speed, and cost‐effectiveness of genetic evaluations. These improvements enhance our ability to assess fetal prognosis and provide more informed counseling to parents. No studies analyzing the role of modern techniques of gene diagnosis like chromosome micro arrays (CMA) or whole exome sequencing within the scope of ocular malformations could be found. Depending on pattern of structural changes, those two techniques offer an increase of diagnostic yield over karyotype alone. In CMA diagnostic gain increases between 5.6% for isolated changes and 13.6% for anomalies affecting multiple organs.^38^ Regarding whole exome sequencing an increase of 31% in diagnostic yield of structurally abnormal fetuses can be achieved when CMA/karyotype is non‐diagnostic^40^. Mellis and colleagues describe in a meta‐analysis, that this increase depends on the selection criteria of the cases ranging between 15% for unselected cases and 42% for pre‐selected cases.^39^

There are limitations to this study inherent to the study design itself. One of the primary limitations of this study is the artificial nature of our cohort, which is heavily influenced by referral patterns and prenatal diagnostic practices. This introduces significant selection bias that may not accurately represent the true population incidence or spectrum of this condition. An indirect selection of particularly complex cases cannot be excluded, and isolated ocular anomalies are probably underrepresented. The limited postmortem examinations of the terminated cases may have missed internal anomalies, potentially underestimating the full spectrum of conditions. Including only confirmed cases might lead to selection bias, possibly overrepresenting more severe or easily identifiable conditions. Due to the retrospective assessment of the cases, there is a substantial risk of missing data. This could lead to underreporting of certain features or outcomes. Although a relatively large cohort was prenatally evaluated, only a few newborns with ocular anomalies were born. This can be explained by the high percentage of termination of pregnancy and cases with perinatal death, most likely related to the higher complexity of associated anomalies. The quality of the images assessed and the range of diagnostic tools have partially changed within the long period of time in which patients were included. This explains why CMA and exome diagnostics were only present in some cases. Some findings like cyclopia, cataracts, and retinal detachment were rarely described; hence, the small size of these groups allowed no statistical analysis describing relevant findings about outcomes or related anomalies. A further limitation of the retrospective design is the limitation of the assessment of the detailed spatial relationship of some structures, depending on static images.

## 5 CONCLUSION

There is a significant association between fetal eye anomalies and certain extraocular anomalies like exophthalmos and cranial bone deformities. A significant association could also be found between genetic aberrations like trisomy 13 and hypotelorism or triploidy and FGFR mutations and exophthalmos. Systematic evaluation of the fetal eye using the proposed protocol is simple to learn and highly reproducible. Data from this study could help to concentrate the diagnostic effort on a certain specific group of malformations and to develop targeted molecular diagnostic tools.

## AUTHOR CONTRIBUTIONS

Jorge Jiménez Cruz and Paul Böckenhoff were responsible for conceptualization, data curation, performing the investigation, formal and statistical analysis, writing the original draft, and editing; Laura Tascón Padrón, Eva Weber, and Norah Emrich were responsible for data acquisition in their centers and reviewed the manuscript; Philipp Kosian contributed to data validation and reviewing the manuscript; Brigitte Strizek, Cristoph Berg, and Ulrich Gembruch were responsible for project administration and supervision, as well as validation of the data in each center, as well as writing the review and editing the manuscript. Brigitte Strizek and Annegret Geipel were involved in conceptualization, formal analysis, development of the methodology, as well as identifying resources and validation of the data, and writing, reviewing, and editing the manuscript. All authors had full access to all the data in the study and had final responsibility for the decision to submit for publication.

## FUNDING INFORMATION

This research was conducted using institutional resources without external funding.

## CONFLICT OF INTEREST STATEMENT

The authors declare no conflicts of interest.

## ETHICS STATEMENT

The Ethics Committee of the University of Bonn does not request formal approval for anonymized retrospective analyses of clinical data.

## Supporting information


**Table S1.** Risk calculation for extraocular changes depending on ultrasound eye findings. Gray marked are statistically significant correlations (*p* < 0.05). *Chi squared test results *p* = 0.04, although 95% CI for OR includes 1.

## References

[1] Ying X , Li H , Yew DT . Morphometric measurements of fetal and neonatal eyes using MRI and ultrasound. Neuroembryol Aging. 2008;5:60‐62.

[2] Bethune M , Alibrahim E , Davies B , Yong E . A pictorial guide for the second trimester ultrasound. Australas J Ultrasound Med. 2013;16:98‐113. 28191183 10.1002/j.2205-0140.2013.tb00106.x PMC5029995

[3] Trout T , Budorick NE , Pretorius DH , McGahan JP . Significance of orbital measurements in the fetus. J Ultrasound Med. 1994;13:937‐943. 7877204 10.7863/jum.1994.13.12.937

[4] Feldman N , Melcer Y , Levinsohn‐Tavor O , et al. Prenatal ultrasound charts of orbital total axial length measurement (TAL): a valuable data for correct fetal eye malformation assessment. Prenat Diagn. 2015;35:558‐563. 25655829 10.1002/pd.4572

[5] Goldstein I , Tamir A , Zimmer EZ , Itskovitz‐Eldor J . Growth of the fetal orbit and lens in normal pregnancies. Ultrasound Obstet Gynecol. 1998;12:175‐179. 9793189 10.1046/j.1469-0705.1998.12030175.x

[6] Merz E , Wellek S , Püttmann S , Bahlmann F , Weber G . Orbitadurchmesser, innerer und äusserer Orbitaabstand. Ein Wachstumsmodell für die fetalen Orbitamasse [Orbital diameter, inner and outer orbital distance. A growth model of fetal orbital measurements] (In German). Ultraschall Med. 1995;16:12‐17. 7709212 10.1055/s-2007-1003230

[7] Jacquemyn Y , Sys SU , Verdonk P . Fetal binocular distance: no differences between ethnic groups. Gynecol Obstet Investig. 2000;50:24‐27. 10895023 10.1159/000010273

[8] Rosati P , Bartolozzi F , Guariglia L . Reference values of fetal orbital measurements by transvaginal scan in early pregnancy. Prenat Diagn. 2002;22:851‐855. 12378563 10.1002/pd.421

[9] Guariglia L , Rosati P . Early transvaginal biometry of fetal orbits: a cross‐sectional study. Fetal Diagn Ther. 2002;17:42‐47. 11803216 10.1159/000048005

[10] Searle A , Shetty P , Melov SJ , Alahakoon TI . Prenatal diagnosis and implications of microphthalmia and anophthalmia with a review of current ultrasound guidelines: two case reports. J Med Case Rep. 2018;12:250. 30153864 10.1186/s13256-018-1746-4 PMC6114735

[11] Ondeck CL , Pretorius D , McCaulley J , et al. Ultrasonographic prenatal imaging of fetal ocular and orbital abnormalities. Surv Ophthalmol. 2018;63:745‐753. 29705173 10.1016/j.survophthal.2018.04.006

[12] Bronshtein M , Zimmer E , Gershoni‐Baruch R , Yoffe N , Meyer H , Blumenfeld Z . First‐ and second‐trimester diagnosis of fetal ocular defects and associated anomalies: report of eight cases. Obstet Gynecol. 1991;77:443‐449. 1992414

[13] Burns NS , Iyer RS , Robinson AJ , Chapman T . Diagnostic imaging of fetal and pediatric orbital abnormalities. Am J Roentgenol. 2013;201:W797‐W808. 24261386 10.2214/AJR.13.10949

[14] Lee A , Deutinger J , Bernaschek G . Three dimensional ultrasound: abnormalities of the fetal face in surface and volume rendering mode. BJOG. 1995;102:302‐306. 10.1111/j.1471-0528.1995.tb09136.x 7612513

[15] Araujo Júnior E , Kawanami TE , Nardozza LMM , Milani HJF , Oliveira PS , Moron AF . Prenatal diagnosis of bilateral anophthalmia by 3D “reverse face” view ultrasound and magnetic resonance imaging. Taiwan J Obstet Gynecol. 2012;51(4):616‐619. doi:10.1016/j.tjog.2012.09.018 23276567

[16] Brémond‐Gignac D , Copin H , Elmaleh M , Milazzo S . Fetal ocular anomalies: the advantages of prenatal magnetic resonance imaging [Anomalies oculaires foetales: apport de l'imagerie anténatale en résonance magnétique] (In French). J Fr Ophtalmol. 2010;33:350‐354. 20451288 10.1016/j.jfo.2010.03.004

[17] Mashiach R , Vardimon D , Kaplan B , Shalev J , Meizner I . Early sonographic detection of recurrent fetal eye anomalies. Ultrasound Obstet Gynecol. 2004;24:640‐643. 15517557 10.1002/uog.1748

[18] AIUM practice parameter for the performance of detailed diagnostic obstetric ultrasound examinations between 12 weeks 0 days and 13 weeks 6 days. J Ultrasound Med. 2021;40:E1‐E16. 32852128 10.1002/jum.15477

[19] International Society of Ultrasound in Obstetrics and Gynecology , Bilardo CM , Chaoui R , et al. ISUOG practice guidelines (updated): performance of 11–14‐week ultrasound scan. Ultrasound Obstet Gynecol. 2023;61:127‐143. 36594739 10.1002/uog.26106

[20] von Kaisenberg C , Chaoui R , Häusler M , et al. Quality requirements for the early fetal ultrasound assessment at 11‐13+6 weeks of gestation (DEGUM levels II and III). Ultraschall Med. 2016;37:297‐302. 27093520 10.1055/s-0042-105514

[21] Mizia K , Campbell Westerway S , Robertson M , et al. Guidelines for the performance of the first trimester ultrasound. Australas J Ultrasound Med. 2018;21(3):179‐182. doi:10.1002/ajum.12102 34760519 PMC8409877

[22] van den Hof MC , Smithies M , Nevo O , Oullet A . No. 375‐clinical practice guideline on the use of first trimester ultrasound. J Obstet Gynaecol Can. 2019;41:388‐395. 30784569 10.1016/j.jogc.2018.09.020

[23] Merz E , Eichhorn K‐H , Von Kaisenberg C , Schramm T . Aktualisierte Qualitätsanforderungen an die weiterführende differenzierte Ultraschalluntersuchung in der pränatalen Diagnostik (= DEGUM‐Stufe II) im Zeitraum von 18 + 0 bis 21 + 6 Schwangerschaftswochen [updated quality requirements regarding secondary differentiated ultrasound examination in prenatal diagnostics (= DEGUM level II) in the period from 18 + 0 to 21 + 6 weeks of gestation] in German. Ultraschall Med. 2012;33:593‐596. 23147272 10.1055/s-0032-1325500

[24] Cargill Y , Morin L . No. 223‐content of a complete routine second trimester obstetrical ultrasound examination and report. J Obstet Gynaecol Can. 2017;39:e144‐e149. 28729106 10.1016/j.jogc.2017.04.022

[25] ASUM (Australasian Society for Ultrasound in Medicine) . Guidelines for the Performance of Second (Mid) Trimester Ultrasound. Accesesd February 22, 2018. https://www.asum.com.au/standards‐of‐practice/obstetrics‐and‐gynaecology/

[26] Pilu G , Nicolaides KH . Diagnosis of Fetal Abnormalities, the 18‐23‐Week Scan. Vol 1. CRC Press; 2019.

[27] American Institute of Ultrasound in Medicine . AIUM practice parameter for the performance of detailed second‐ and third‐trimester diagnostic obstetric ultrasound examinations. J Ultrasound Med. 2019;38:3093‐3100. 31736130 10.1002/jum.15163

[28] Salomon LJ , Alfirevic Z , Berghella V , et al. ISUOG practice guidelines (updated): performance of the routine mid‐trimester fetal ultrasound scan. Ultrasound Obstet Gynecol. 2022;59:840‐856. 35592929 10.1002/uog.24888

[29] Committee on Practice Bulletins—Obstetrics and the American Institute of Ultrasound in Medicine . Practice bulletin No. 175: ultrasound in pregnancy. Obstet Gynecol. 2016;128:e241‐e256. 27875472 10.1097/AOG.0000000000001815

[30] Papp C , Beke A , Ban Z , Szigeti Z , Toth‐Pal E , Papp Z . Prenatal diagnosis of trisomy 13: analysis of 28 cases. J Ultrasound Med. 2006;25:429‐435. 16567430 10.7863/jum.2006.25.4.429

[31] Menashe Y , Ben Baruch G , Rabinovitch O , Shalev Y , Katzenlson MB , Shalev E . Exophthalmus—prenatal ultrasonic features for diagnosis of Crouzon syndrome. Prenat Diagn. 1989;9:805‐808. 2694154 10.1002/pd.1970091109

[32] Proisy M , Riffaud L , Chouklati K , Tréguier C , Bruneau B . Ultrasonography for the diagnosis of craniosynostosis. Eur J Radiol. 2017;90:250‐255. 28583642 10.1016/j.ejrad.2017.03.006

[33] Berceanu C , Gheonea IA , Vlădăreanu S , et al. Ultrasound and MRI comprehensive approach in prenatal diagnosis of fetal osteochondrodysplasias. Cases series. Med Ultrason. 2017;19:66‐72. 28180199 10.11152/mu-922

[34] Massalska D , Bijok J , Ilnicka A , Jakiel G , Roszkowski T . Triploidy—variability of sonographic phenotypes. Prenat Diagn. 2017;37:774‐780. 28573747 10.1002/pd.5080

[35] Sergi C , Schiesser M , Adam S , Otto HF . Analysis of the spectrum of malformations in human fetuses of the second and third trimester of pregnancy with human triploidy. Pathologica. 2000;92:257‐263. 11029886

[36] Iijima S , Ohzeki T . Extremely low birthweight infant with wolf‐hirschhorn syndrome: a dilemma in determination of the optimal timing of delivery. Clin Med Case Rep. 2008;1:37‐40. 24179343 10.4137/ccrep.s760 PMC3785355

[37] Lueder GT . Clinical ocular abnormalities in infants with trisomy 13. Am J Ophthalmol. 2006;141:1057‐1060. 16765673 10.1016/j.ajo.2005.12.048

[38] Donnelly JC , Platt LD , Rebarber A , Zachary J , Grobman WA , Wapner RJ . Association of copy number variants with specific ultrasonographically detected fetal anomalies. Obstet Gynecol. 2014;124:83‐90. 24901266 10.1097/AOG.0000000000000336 PMC4111105

[39] Mellis R , Oprych K , Scotchman E , Hill M , Chitty LS . Diagnostic yield of exome sequencing for prenatal diagnosis of fetal structural anomalies: a systematic review and meta‐analysis. Prenat Diagn. 2022;42:662‐685. 35170059 10.1002/pd.6115 PMC9325531

