# Supplementary material for: Sonographic diagnosis of fetal eye anomalies and their association with syndromal diseases: A retrospective multicenter analysis of 264 cases
Source: Acta Obstet Gynecol Scand. 2025 Mar 4;104(5):850–9. doi: 10.1111/aogs.15085 (PMC11981110; doi:10.1111/aogs.15085)
Supplement: Supplementary file 1 — Table S1. Risk calculation for extraocular changes depending on ultrasound eye findings. Gray marked are statistically significant correlations (p < 0.05). *Chi squared test results p = 0.04, although 95% CI for OR includes 1. [file AOGS-104-850-s001.docx]

**Table S1: Risk calculation for extraocular changes depending on ultrasound eye findings.**

| **Extraocular defects** | **Anophthalmia** | **Exophthalmos** | **Hypertelorism** | **Hypotelorism** | **Cataract** | **Microphthalmia** | **Retinal detachment** | **Cyclopia** |
| --- | --- | --- | --- | --- | --- | --- | --- | --- |
| Nervous System | 2 (0.6-6.2) | **0.3 (0.1-0.6)** | 0.6 (0.3-1.1) | **2.2 (1.2-4.1)** | 1 (0.4-2.6) | 1.3 (0.7-2.4) | 1.3 (0.3-6.7) | 3.1 (0.3-26.5) |
| Cranial anatomy | 1.3 (0.5-3.4) | **3.3 (1.4-7.4)** | 1.7 (0.9-2.9) | 0.8 (0.5-1.5) | **0.2 (0.1-0.8)** | **0.4 (0.2-0.7)** | 0.4 (0.1-2.4) | 3 (0.6-6.2) |
| Limbs | **0.3 (0.1-0.9)** | 1.9 (0.9-4.2) | **2.1 (1.1-3.7)** | 1.1 (0.6-1.9) | 1.3 (0.5-3.1) | 0.7 (0.4-1.2) | **0.5 (0.5-0.6)** | 0.9 (0.2-3.9) |
| Heart | 0.7 (0.2-1.7) | 1.4 (0.7-3.1) | 0.7 (0.4-1.3) | 1.6 (0.9-2.8) | 1.6 (0.6-3.9) | 0.8 (0.4-1.4) | 0.4 (0.1-2.4) | 2.9(0.6-15.5) |
| Cleft | 1.7 (0.7-4.5) | **0.2 (0.1–0.7)** | 1.1 (0.6-2) | 1.7 (0.9-3.1) | 0.7 (0.2-2) | 0.7 (0.4-1.4) | 0.4 (0.1-3.2) | 1.7 (0.4-7.9) |
| Umbilical cord | 0.6 (0.1-2.8) | 1.8 (0.7-4.4) | 1 (0.5-2.1) | 0.4 (0.2-1) | 0.8 (0.2-2.8) | 1.2 (0.6-2.5) | 0.9 (0.9-1) | **7 (1.5-32.1)** |
| Spinal tract | 1.9 (0.4-9.1) | **5.3 (1.8-15.7)** | 2 (0.7-5.8) | 0.6 (0.2-2.2) | 0.9 (0.9-1) | **0.7 (0.6-0.8)** | 0.9 (0.9-1) | 0.9 (0.9-1) |
| Gastrointestinal tract | **3.6 (1.3-10.5)** | 1.3 (0.4-3.5) | 0.6 (0.2-1.6) | 1.5 (0.7-3.1) | 2.7 (0.-11.1) | 0.6 (0.2-1.5) | 0.9 (0.9-1) | 1.1 (0.1-9.1) |
| Abdominal wall | 2.9 (0.9-9.7) | 0.9 (0.9-1) | 1.3 (0.5-3.4) | 1.1 (0.3-3.8) | 0.9 (0.9-1) | 0.8 (0.3-2.2) | 0.9 (0.9-1) | 1.7 (0.2-14.5) |
| Neck | 0.5 (0.1-4.1) | 2.1 (0.7-6.1) | 0.8 (0.3-2.3) | 1.1 (0.5-2.8) | 1 (0.3-4.7) | 0.6 (0.2-1.7) | 0.9 (0.9-1) | 4.2 (0.8-22.9) |
| Chest | 1.6 (0.4-5.8) | **3.6 (1.5-8.8)** | 1.3 (0.6-2.9) | 0.5 (0.2-1.2) | 0.8 (0.5-5.6) | **0.3 (0.1-0.9)** | 1.2 (0.2-10.2) | 0.5 (0.1-4.5) |
| Renal system | 0.6 (0.2-2.3) | 0.6 (0.2-1.6) | 0.9 (0.5-1.9) | 1 (0.5-1.9) | 1.9 (0.7-5) | **2.3 (1.2-4.3)** | 0.5 (0.1-4.5) | 0.9 (0.9-1) |
| Skeletal system | 1.8 (0.4-8.4) | **4.8 (1.6-14)** | 1.8 (0.6-5.1) | 0.2 (0.02-1.2) | 0.7 (0.1-5.5) | 0.4 (0.1-1.8) | 0.9 (0.9-1) | 0.9 (0.9-1) |
| Aneuploidy | 1.1 (0.4-3.4) | **0.3 (0.1-0.9)** | **0.5 (0.2-0.9)** | **3.4 (1.8-6.3)** | 1 (0.4-2.8) | 1.3 (0.7-3.5) | 0.4 (0.04-3.6) | **8.3 (0.9-76.1)*** |
| Gray marked are statistically significant correlations (p < 0.05)  *Chi Squared Test results p = 0.04, although 95% CI for OR includes 1 | | | | | | | | |
